# The Apolipoprotein A-I Mimetic L-4F Attenuates Monocyte Activation and Adverse Cardiac Remodeling after Myocardial Infarction

**DOI:** 10.3390/ijms21103519

**Published:** 2020-05-15

**Authors:** Tariq Hamid, Mohamed Ameen Ismahil, Shyam S. Bansal, Bindiya Patel, Mehak Goel, C. Roger White, G. M. Anantharamaiah, Sumanth D. Prabhu

**Affiliations:** 1 Division of Cardiovascular Disease, Department of Medicine, University of Alabama at Birmingham, Birmingham, AL 35233, USA; ameenismahil@uabmc.edu (M.A.I.); shyam.bansal@osumc.edu (S.S.B.); bindiyapatel01@gmail.com (B.P.); mehakgoel.bph@gmail.com (M.G.); rogerwhite@uabmc.edu (C.R.W.); 2 Division of Gerontology, Geriatrics, and Palliative Care, Department of Medicine, University of Alabama at Birmingham, Birmingham, AL 35233, USA; ganantha@uabmc.edu; 3 Medical Service, Birmingham VAMC Birmingham, Birmingham, AL 35233, USA

**Keywords:** inflammation, heart failure, cardiac remodeling, macrophage polarity

## Abstract

Excessive inflammation after myocardial infarction (MI) can promote infarct expansion and adverse left ventricular (LV) remodeling. L-4F, a mimetic peptide of apolipoprotein A-I (apoA-I), exhibits anti-inflammatory and anti-atherogenic properties; however, whether L-4F imparts beneficial effects after myocardial infarction (MI) is unknown. Here we demonstrate that L-4F suppresses the expansion of blood, splenic, and myocardial pro-inflammatory monocytes and macrophages in a mouse model of reperfused MI. Changes in immune cell profiles were accompanied by alleviation of post-MI LV remodeling and dysfunction. In vitro, L-4F also inhibited pro-inflammatory and glycolytic gene expression in macrophages. In summary, L-4F treatment prevents prolonged and excessive inflammation after MI, in part through modulation of pro-inflammatory monocytes and macrophages, and improves post-MI LV remodeling. These data suggest that L-4F could be a used as a therapeutic adjunct in humans with MI to limit inflammation and alleviate the progression to heart failure.

## 1. Introduction

Healing after myocardial infarction (MI) occurs in a biphasic manner [1]. Initially, there is an intense inflammatory response characterized by robust infiltration of neutrophils and subsequently bone marrow- and spleen-derived monocytes and macrophages that promote the digestion and clearance of necrotic myocardial tissue [2,3]. This is then followed by inflammation resolution, marked by a shift in tissue macrophage polarity towards a reparative phenotype that facilitates tissue neovascularization and scar formation. An inflammatory response that is excessive or fails to resolve effectively can lead to chronic inflammation with persistence of pro-inflammatory macrophages and T-cells that perpetuate tissue injury, leading to adverse cardiac remodeling and heart failure (HF) [4,5,6]. Hence, suppressing pro-inflammatory macrophage responses while facilitating reparative macrophages following MI represents a potential therapeutic approach to limit pathology and prevent HF. To date, however, there are no extant immunomodulatory therapies in humans to favorably alter macrophage responses for therapeutic benefits after MI.

L-4F is a peptide mimetic of apolipoprotein A-I (apoA-I), the major protein component of high-density lipoprotein (HDL) [7,8]. The structure of L-4F is based on tandem repeating amphipathic helical domains present in naturally occurring human apoA-I. L-4F has been demonstrated to exert robust anti-inflammatory, anti-oxidant, and anti-atherogenic effects in several animal models [7,8,9,10,11,12]. The anti-inflammatory effects may relate to modulation of innate immune cells, as in vitro, L-4F promotes M2 differentiation of monocyte-derived macrophages and inhibits their endothelial adhesion and migration [13]. In view of these in vitro responses, we tested the hypothesis that L-4F would favorably impact circulating monocytes and tissue macrophages in vivo after MI, thereby imparting beneficial effects on cardiac healing. Our results demonstrate for the first time that administration of L-4F after the acute post-MI inflammatory phase suppresses pro-inflammatory monocytes and macrophages in the blood, spleen, and heart, and alleviates post-MI cardiac remodeling. In addition, L-4F inhibited glycolytic and pro-inflammatory gene expression in M1-polarized macrophages without significantly impacting M2-polarized cells in vitro, thereby promoting a reparative phenotype.

## 2. Results

### 2.1. L-4F Attenuates Adverse Post-MI Left Ventricular (LV) Remodeling and Systolic Dysfunction

**Figure 1:**
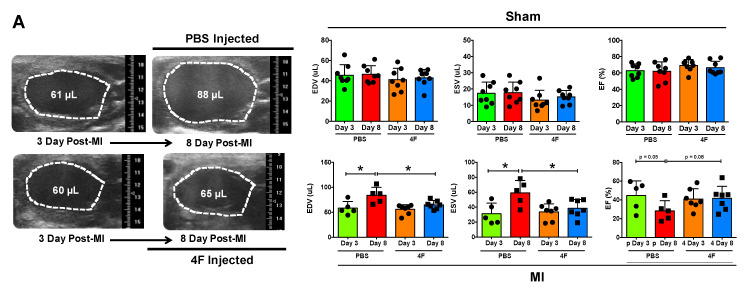

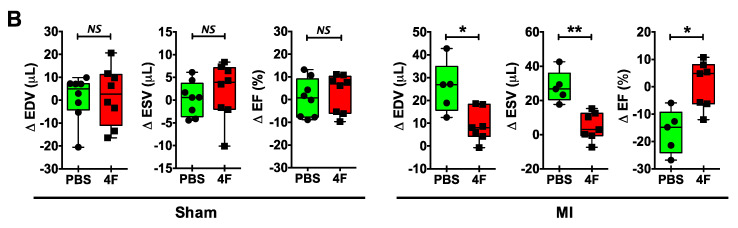
L-4F attenuates left ventricular (LV) remodeling after reperfused myocardial infarction (MI). (**A**) Representative long axis B-Mode echocardiographic images at end-diastole and the corresponding group data. Serial images were acquired at 3- and 8-day post ischemia/reperfusion. Mice were either injected with PBS or 100 μg/day L-4F for 5 days starting at 3-day post-MI. The dotted line marks the LV cavity. (**B**) Echocardiographic group data depicting changes (Δ) in end-diastolic and end-systolic volume (EDV and ESV) and ejection fraction (EF) from 3- to 8-days post-MI or sham operation with treatment with either PBS or L-4F as in (**A**) *n* = 6–8/group, * *p* < 0.05, ** *p* < 0.005. NS – not significant.

We sought to test whether L-4F would beneficially restrain excessive residual inflammation post-MI. Hence, we specifically evaluated its effects after the peak of the post-MI acute inflammatory response required to initiate cardiac wound healing. L-4F or PBS vehicle were given daily starting 3 days after reperfused MI or sham surgery, and left ventricular (LV) remodeling and function were assessed on day 8. Echocardiography of sham-operated mice revealed no differences between initial (pre-treatment) LV end-diastolic and end-systolic volume (EDV and ESV) and LV ejection fraction (EF) (Figure 1A), and no meaningful changes in EDV, ESV, and EF over time after either L-4F or PBS treatment (Figure 1B). MI mice assigned to receive either L-4F or PBS exhibited similar LV EDV, ESV, and EF prior to treatment. However, as compared with PBS-treated MI mice, 5 days of L-4F treatment post-MI abrogated progressive LV chamber dilatation and dysfunction (Figure 1A,B). Analysis of the changes in LV EDV, ESV, and EF after treatment (day 3 to day 8) indicated maintained LV size and EF in L-4F treated MI mice, with significantly smaller changes in these parameters than PBS-treated MI mice. Hence, L-4F therapy prevented the progression of LV remodeling in the subacute period after MI.

### 2.2. L-4F Alleviates Systemic Ly6C^hi^ Monocyte Activation after Reperfused MI

**Figure 2:**
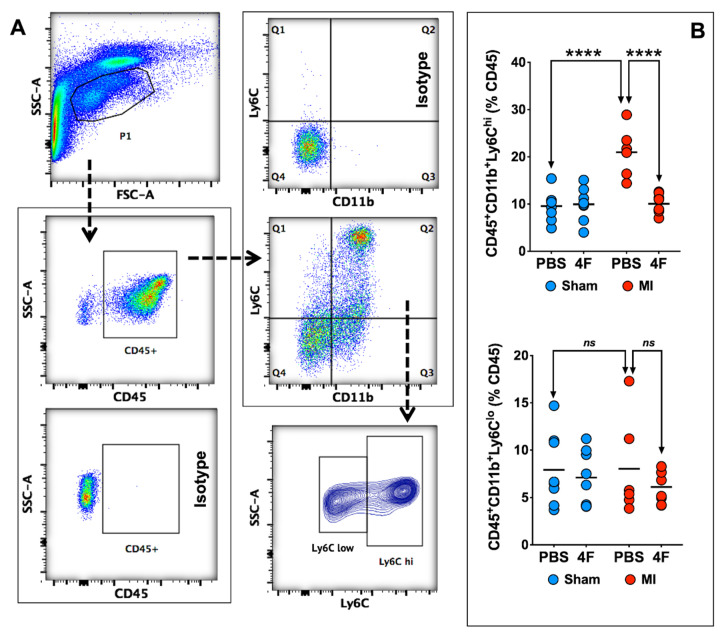
L-4F suppresses blood pro-inflammatory monocyte levels. (**A**) Gating strategy for flow cytometric evaluation of peripheral blood monocytes. CD45^+^ cells within the lymphocyte/monocyte gate (P1) were further gated on the basis of CD11b and Ly6C expression. Pro-inflammatory monocytes were identified as CD45^+^CD11b^+^Ly6C^hi^ cells while patrolling monocytes were identified as CD45^+^Cd11b^+^Ly6C^low^ cells. (**B**) Flow cytometric group data of peripheral blood monocytes after L-4F or PBS treatment post-reperfused MI. Data are depicted as percent CD45^+^ cells. *n* = 5–8/group, **** *p* < 0.0005. ns – not significant.

Figure 2 depicts representative peripheral blood flow cytometry gates for identification of pro-inflammatory Ly6C^hi^ and patrolling Ly6C^low^ monocytes, and corresponding quantitation of cell frequency after 5 days of treatment with either L-4F or PBS in MI and sham mice as described above. In sham mice, circulating levels of both Ly6C^hi^ and Ly6C^low^ monocytes were comparable regardless of treatment group. In PBS-treated MI mice, as would be anticipated, Ly6C^hi^ monocytes were significantly increased nearly 3-fold as compared with sham-PBS (or sham-L-4F) mice, without observed differences in Ly6C^low^ monocytes. Notably, L-4F markedly suppressed Ly6C^hi^ monocytosis in MI mice, such that levels were comparable to those observed in sham mice. There were no significant L-4F-mediated effects on circulating Ly6C^low^ monocytes in MI mice.

**Figure 3:**
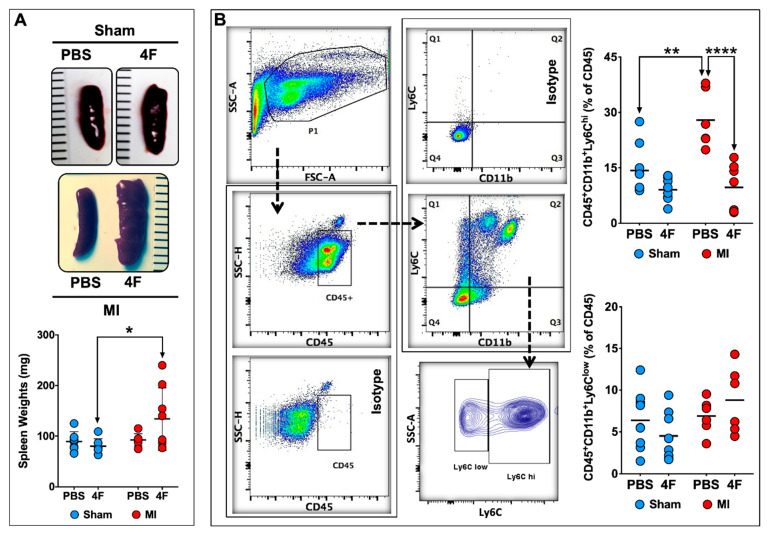
L-4F suppresses levels of pro-inflammatory splenic monocytes post-MI. (**A**) Representative gross images of spleens harvested from sham-operated or MI mice injected with either PBS or L-4F (100 μg/day) for 5 days starting at day 3 post-surgery, and the corresponding gravimetric quantitation. (**B**) Gating strategy and group data for flow cytometric quantitation of splenic monocytes following L-4F or PBS treatment. Splenic pro-inflammatory and patrolling monocytes were identified as CD45^+^CD11b^+^Ly6C^hi^ and CD45^+^CD11b^+^Ly6C^low^ cells, respectively. Data are depicted as percent of CD45^+^ cells, *n* = 5–8/group. ** *p* < 0.005, **** *p* < 0.0005.

As the spleen is an important source of circulating monocytes and monocyte-derived infiltrating tissue macrophages after acute MI [14,15], we next evaluated the effects of L-4F on the spleen at the same time point as in Figure 2. L-4F augmented spleen weight in MI mice as compared with L-4F-treated sham-operated mice (Figure 3A). Representative flow cytometry gates for splenic monocytes and corresponding quantitation of Ly6C^hi^ and Ly6C^low^ monocytes are shown in Figure 3B. Analogous to blood monocytes, PBS-treated MI mice exhibited significantly increased (~2-fold) frequency of pro-inflammatory Ly6C^hi^ monocytes in the spleen at 8-day post-MI (as compared with sham mice), which was markedly suppressed and normalized in L-4F-treated MI mice. Hence, pro-inflammatory blood and splenic monocytes were particularly sensitive to L-4F and were impacted in parallel, suggesting that L-4F reduced abundance and trafficking of splenic Ly6C^hi^ monocytes to the heart after MI. These effects may in part underlie the reported anti-inflammatory properties of L-4F in vivo.

We evaluated the effect of L-4F on splenic extramedullary hematopoiesis as a potential explanation for the splenic hypertrophy observed after L-4F treatment in MI mice. Naïve mice were given L-4F or PBS as above. Isolated splenic cell-suspensions were then analyzed by flow cytometry to quantitate hematopoietic stem cells (HSCs [16]; Lineage (Lin)^−^c-Kit^+^Sca1^+^), common myeloid progenitors (CMPs [17]; Lin^−^c-Kit^+^Sca1^−^), and macrophage dendritic cell progenitors (MDPs [16]; Lin^−^CD115^+^c-Kit^low^). The representative flow cytometry gating strategy to identify HSCs, CMPs, and MDPs and the corresponding group data are depicted in Figure S1. There were no changes in absolute levels of CMPs and MDPs. However, 5 days of L-4F treatment in naïve mice significantly (~2.3 fold) increased the abundance of HSCs in the spleen. Although we did not evaluate splenic progenitors in L-4F-treated MI mice, it has been shown previously that MI independently augments splenic extramedullary monocytopoiesis [15]. These results suggest that L-4F and MI synergistically augment splenic extramedullary hematopoiesis to induce splenic hypertrophy at 8 days post-MI.

### 2.3. L-4F Restrains Pro-Inflammatory Ly6C^hi^ Macrophages in Healing Infarcted Hearts

**Figure 4:**
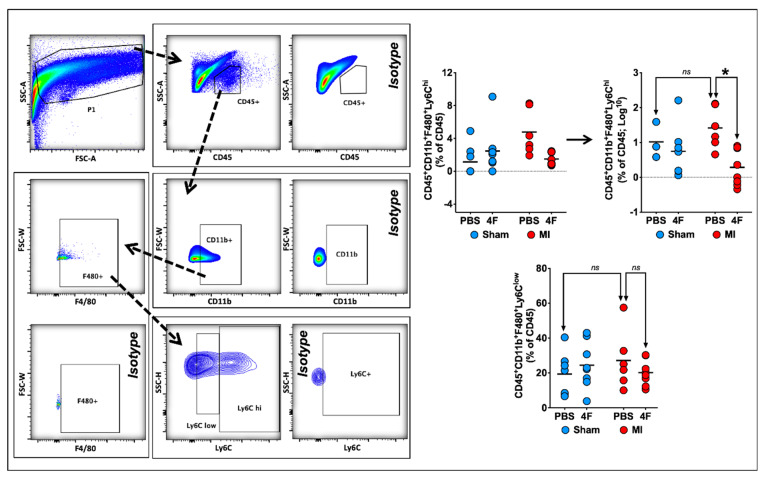
L-4F decreases myocardial pro-inflammatory macrophage expansion in infarcted hearts. Left panel: Gating strategy for flow cytometric evaluation of cardiac macrophages. Right panel: Quantitative group data for macrophage abundance in sham-operated and infarcted hearts after PBS or L-4F treatment for 5 days, starting at 3-day post-surgery. Statistical comparisons were performed after logarithmic data transformation to satisfy the normality assumption as described in the text. Data are presented as percent CD45^+^ cells by flow cytometry, *n* = 6–8/group, * *p* < 0.05. ns—not significant.

Previous studies have demonstrated that both the initial wave of pro-inflammatory (Ly6C^hi^) macrophages and the later predominance of reparative (Ly6C^low^) macrophages in the heart after MI are derived from infiltrating pro-inflammatory Ly6C^hi^ monocytes [18]. Hence, we next examined the effects of delayed L-4F administration post-MI on cardiac macrophages during the healing phase. Figure 4 depicts representative flow cytometry gates to identify F4/80^+^Ly6C^hi^ pro-inflammatory and F4/80^+^Ly6C^low^ reparative macrophages from the cardiac CD45^+^ cell gate. F4/80^+^ cells represented a majority (~65–90%) of the CD11b^+^ myeloid cell population at 8 days post-MI. Analysis of Ly6C^hi^ and Ly6C^low^ macrophage subpopulations in post-MI hearts indicated an L-4F suppressive effect on macrophages limited to pro-inflammatory F4/80^+^Ly6C^hi^ cells, without significant effects on F4/80^+^Ly6C^low^ cells. L-4F had no effect on macrophages in sham-operated hearts. Taken together, these data suggest that L-4F curbed infiltration of Ly6C^hi^ pro-inflammatory macrophages in the infarcted heart and/or promoted tissue macrophage polarization to a M2-type reparative phenotype.

### 2.4. L-4F Inhibits M1-Macrophage Activation and Induces Macrophage Plasticity In Vitro

**Figure 5:**
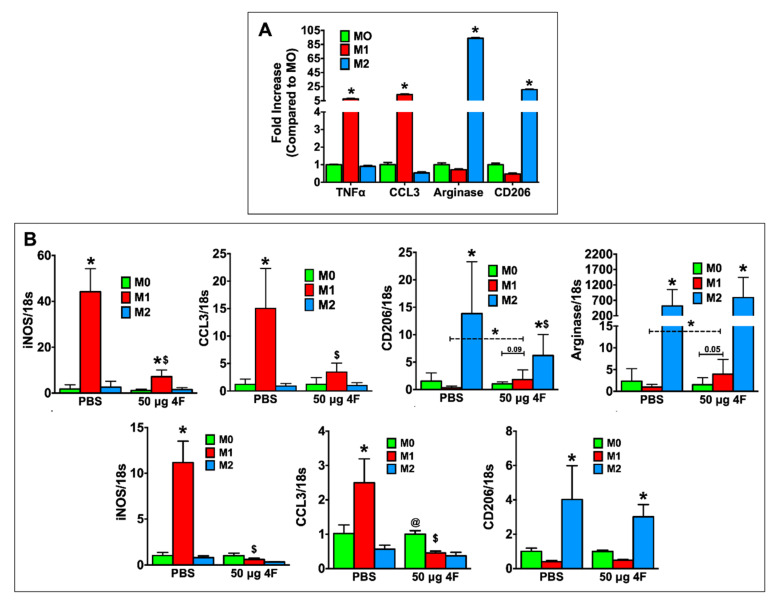
L-4F inhibits M1-macrophage activation and induces macrophage plasticity in vitro. (**A**) RT-PCR analysis of M1 and M2 macrophage-associated marker genes in mouse peritoneal macrophages polarized in vitro. (**B**) RT-PCR analysis of M1 and M2 macrophage-associated marker genes from polarized RAW 264.7 cells (upper panels) or polarized mouse peritoneal macrophages (lower panels) after overnight treatment with either PBS or L-4F (50 μg/mL). Data are presented as fold changes (mean ± SD) and compared to non-polarized M0 macrophages. * *p* < 0.05 vs. respective M0; ^$^ *p* < 0.05 vs. PBS-treated M1 group, ^@^ *p* < 0.05 vs. respective M1 and M2 groups, *n* = 3/group each done in triplicate for RAW 264.7 cells. Thioglycolate-elicited mouse peritoneal macrophages were isolated and pooled from 6 mice and also run in triplicate.

L-4F-induced effects on macrophage polarization in vitro were evaluated in thioglycolate-elicited peritoneal macrophages from C57BL/6 mice and in RAW 264.7 mouse macrophages. For these studies, we sought to define the effects of L-4F on macrophages already differentiated to a pro-inflammatory (M1) or reparative (M2) phenotype to better understand the in vivo effects of L-4F on analogous macrophage populations in the infarcted heart. Therefore, peritoneal macrophages were polarized to either an M1 or M2 phenotype and subsequently treated with either L-4F or PBS. Undifferentiated M0 cells served as controls. As shown in Figure 5A, as compared with M0 macrophages, M1-polarized macrophages exhibited upregulation of pro-inflammatory mediators (tumor necrosis factor α (*TNFα*), C-C motif chemokine ligand 3 [*CCL3*]), whereas M2-polarized macrophages exhibited the expected upregulation of reparative markers (*arginase*, *CD206*), indicating robust and appropriate polarization. Figure 5B depicts gene expression in PBS- and L-4F-treated M0-, M1-, and M2-polarized RAW 264.7 cells (top panels) and peritoneal macrophages (bottom panels). As compared with M0 macrophages, PBS-treated M1 macrophages upregulated inducible nitric oxide synthase (*iNOS*) and *CCL3* expression, whereas L-4F treatment significantly reduced expression of these M1 markers. Moreover, in RAW 264.7 M1-polarized cells when compared specifically to M0-polarized cells, L-4F significantly augmented expression of M2 markers *CD206* and *arginase*.

**Figure 6:**
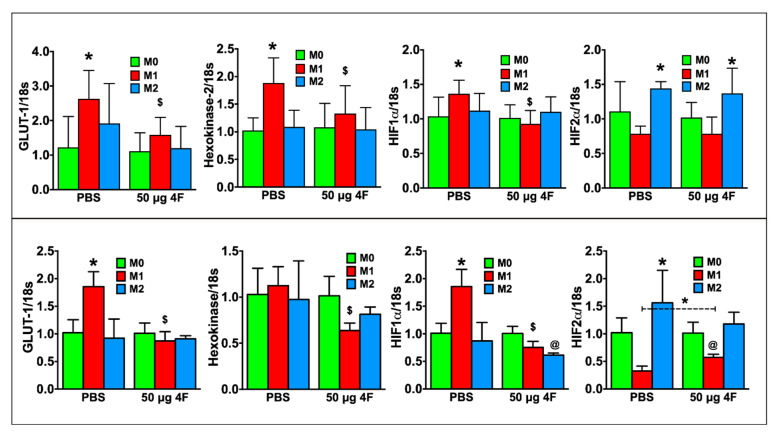
L-4F attenuates M1 macrophage-associated metabolic gene profile in polarized macrophages. RT-PCR analysis of M1-macrophage associated metabolic genes from polarized RAW 264.7 cells (upper panels) or polarized mouse peritoneal macrophages (lower panels) after overnight treatment with either PBS or L-4F (50 μg/mL). Data are presented as fold changes (mean ± SD) and compared to non-polarized M0 macrophages. * *p* < 0.05 vs. respective M0; ^$^ *p* < 0.05 vs. PBS-treated M1 group, ^@^ *p* < 0.05 vs. respective M0 and M2 groups, *n* = 3/group each done in triplicate as in Figure 5.

M1 macrophage polarity is characterized by glycolytic metabolism and hypoxia inducible factor (HIF)-1α isoform expression in contrast to oxidative metabolism and *HIF-2α* isoform expression in M2 macrophages [19,20]. Figure 6 depicts gene expression in RAW 264.7 cells (top panel) and peritoneal macrophages (bottom panel). As compared to M0 macrophages, PBS-treated M1 macrophages exhibited significantly increased expression of the glycolytic markers’ glucose transporter-1 (*GLUT-1*) and/or *hexokinase-2*, as well as *HIF-1α*. *HIF-2α* expression, as anticipated, was significantly increased in PBS-treated M2 macrophages. L-4F treatment significantly downregulated glycolytic gene and *HIF-1α* expression in M1-polarized cells, and also augmented *HIF-2α* expression in M1-polarized peritoneal macrophages. L-4F did not affect *HIF-2α* expression in M2-polarized macrophages. Hence, taken together, L-4F suppressed a pro-inflammatory and glycolytic gene profile and promoted a more reparative profile in M1 macrophages, suggesting L-4F-mediated plasticity of macrophage phenotype.

## 3. Discussion

Herein we show for the first time that treatment post-MI with the apoA-I peptide mimetic L-4F, when initiated after the peak inflammatory response in a clinically-relevant I/R model, suppressed pro-inflammatory monocytes and macrophages and had beneficial effects on post-MI cardiac remodeling. L-4F reduced Ly6C^hi^ monocyte levels in the blood and splenic reservoir, and specifically restrained pro-inflammatory Ly6C^hi^ macrophages in the remodeling heart, while preventing progressive LV dilatation and systolic dysfunction post-MI. In vitro studies suggest that L-4F induces plasticity of macrophage phenotype towards a more reparative profile. Taken together, these studies indicate that targeted immune cell modulation by L-4F yields salutary effects on the infarcted heart. Importantly, L-4F has already been shown to have a sound safety profile in humans [21]; hence, these data also provide strong impetus for testing such an approach in clinical studies.

ApoA-I is the major component of the plasma HDL that mediates cholesterol efflux via reverse cholesterol transport from cells back to the liver [22]. Decreased levels of HDL-cholesterol are directly related to coronary heart disease incidence and mortality in humans, whereas infusion of HDL following myocardial ischemia in mice reduces infarct size and improves cardiac function [23]. apoA-I level is a biomarker for prediction of cardiovascular disease [24,25] and apoA-I gene therapy has been used as a HDL-raising strategy. Studies of human apoA-I gene transfer in mice resulted in improved survival and cardiac remodeling and function post-MI [26,27]. These apoA-1-mediated effects are primarily attributed to decreases in myocardial apoptosis and oxidative stress [27]. However, whether HDL-raising directly affects immune cell activation and function is not known. L-4F is an apoA-I mimetic peptide that exhibits anti-atherogenic, anti-inflammatory, and antioxidant effects [9,10,11,12] via binding and inhibition of pro-inflammatory oxidized lipids [9]. 4F also inhibits lipopolysaccharide-induced expression of pro-inflammatory cytokines in neutrophils, reduces vascular monocytic adhesion, decreases platelet aggregation [28], and promotes an anti-inflammatory phenotype in human monocyte-derived macrophages [13,29]. L-4F-induced differentiation of monocyte-derived macrophages towards an anti-inflammatory state was recently shown to occur as a result of induction of an oxidative metabolic program and increase in macrophage fatty acid uptake [30]. Long-term L-4F treatment has also been previously reported to have beneficial cardiac functional effects in a genetic type II diabetes mouse model, which were attributed to decreased systemic pro-inflammatory cytokines and increased myocardial expression of heme oxygenase-1 [31].

Human clinical trials of 4F peptides to reduce atherosclerotic risk have not yet yielded encouraging results [7,8,21,32]; nonetheless, the robust anti-inflammatory effects of L-4F suggest that it may yield therapeutic benefit as an immune cell modulator in conditions of heightened inflammation or impaired resolution. Given the critical importance of phasic changes in macrophage phenotype required for effective wound healing and remodeling after MI [1,2], we tested the effects of time-limited administration of L-4F post-MI on monocyte/macrophage populations and cardiac structure and function. In most medical scenarios, patients with acute ST-elevation MI are treated expeditiously with acute coronary reperfusion. To mimic the clinical situation, we employed a murine model of 60 min of total coronary occlusion followed by reperfusion. After non-reperfused MI, the acute inflammatory phase peaks at day ~3–4 post-MI, with the reparative phase peaking by day ~7 [1,2]. Coronary reperfusion results in an earlier peak and faster decline of inflammatory cell infiltration [3]. As the initial innate immune response is essential for the fidelity of subsequent repair [33,34], we chose to administer L-4F starting at 3 days post-MI to avoid suppressing this critical early inflammatory surge required for subsequent healing. The key finding of our study is that time-targeted restraint of pro-inflammatory monocyte/macrophages in this manner by 4-LF was accompanied by alleviation of subsequent pathological LV remodeling.

Recent studies indicate that while pro-inflammatory M1-type macrophages dominate the initial inflammatory phase of the tissue response post-MI and reparative M2-type macrophages govern the subsequent resolution and healing phase, both of these macrophage populations are fundamentally derived from infiltrating Ly6C^hi^ monocytes [18]. Moreover, time-appropriate suppression of inflammatory macrophage profiles and/or induction of reparative macrophage polarity may represent an effective therapeutic post-MI intervention to prevent subsequent HF. Circulating monocytes that are recruited to the infarcted heart originate from bone marrow and splenic reservoirs [14]. In this regard, it is important to note that the pro-inflammatory monocyte/macrophage modulation by L-4F occurred at both local and systemic levels, as L-4F globally suppressed Ly6C^hi^ cells in the spleen, blood and infarcted heart without altering tissue reparative macrophages. The monocyte profile after MI was favorably altered by L-4F despite augmented extramedullary hematopoiesis in the spleen.

Previously, it was shown that primary monocytes exposed to L-4F downregulated their expression of classical activation markers (e.g., HLA-DR, CD14, CD11b) and instead evidenced features of alternative activation, related in part to increased mitochondrial fatty acid uptake and oxidative metabolism [13,30]. We now extend this prior work by demonstrating the effects of L-4F on macrophages already committed to classical or alternative phenotypes as would be expected to occur in the healing post-MI heart. Our results indicate that L-4F primarily and selectively modulates committed M1-type macrophages by inducing downregulation of classical M1-associated inflammatory genes (e.g., *iNOS*, *CCL3*), typical glycolytic markers [19], and *HIF-1α* [20], as well as upregulating aspects of the M2 gene profile (e.g., *CD206*, *arginase*, *HIF-2α*). Hence, L-4F induces plasticity of macrophage phenotype that in the healing heart would potentially favor inflammation resolution and tissue repair. Although not directly assessed in our studies, L-4F could also be imparting its immunomodulatory effects by affecting platelet–monocyte interactions. Platelet–monocyte complexes are increased in acute MI [35] and failing hearts [36], and L-4F is known to inhibit platelet aggregation [28].

In summary, we have established that time-targeted administration of the apoA-I mimetic L-4F after MI effectively suppresses the pro-inflammatory monocyte and macrophage response systemically and locally in the heart, and imparts beneficial effects on subsequent cardiac remodeling. Moreover, L-4F has the capacity to induce plasticity in pro-inflammatory M1-type macrophages towards a more resolving and reparative phenotype. As L-4F has a well-established and acceptable safety profile in humans, these results support the testing of such an approach to modulate macrophage biology, limiting chronic inflammation and the development of ischemic cardiomyopathy after MI.

## 4. Materials and Methods

All studies were performed in compliance with the NIH Guide for the Care and Use of Laboratory Animals (DHHS publication No. [NIH] 85–23, revised 1996) and The University of Alabama at Birmingham Institutional Animal Care and Use Committee.

### 4.1. L-4F Peptide Synthesis and Purification

L-4F peptide was synthesized using a solid-phase peptide synthesis method as described elsewhere [37]. The synthesized peptide was purified using reverse phase HPLC and the purity was confirmed by analytical HPLC and mass spectral analysis.

### 4.2. Mouse MI Model and Experimental Protocol

Male C57BL/6J mice 10–12 weeks of age were used for these studies. Acute MI was induced by subjecting mice to open-chest left coronary artery ligature occlusion and reperfusion (I/R) under 1.5%–2.0% isoflurane inhalation anesthesia as described previously [38,39], but with an ischemic time of 60 min followed by extended reperfusion. Sham-operated mice served as controls. Mice were assessed at day 3 post-MI by echocardiography, and mice with similar degrees of myocardial injury (or sham control mice) were randomized to receive either L-4F (100 μg/day in 200 μL of PBS) or PBS vehicle (200 μL/injection) via the tail vein for 5 consecutive days. This dose of L-4F was based on a previous study evaluating the efficacy of L-4F in inhibiting LDL aggregation [40]. After 5 days of treatment, echocardiography was repeated, peripheral blood was collected, and the mice were sacrificed and tissue processed for immune cell analysis by flow cytometry. The project was approved by The University of Alabama at Birmingham Institutional Animal Care and Use Committee (IACUC) on 3 October 2017 (IACUC-21091).

### 4.3. Echocardiography

Echocardiography was performed under anesthesia with tribromoethanol (0.25 mg/g IP), and light (1–2%) isoflurane as needed, using a VisualSonics Vevo 770 High-Resolution System with a RMV707B scan head as previously described [5,41]. The mice were imaged on a heated bench-mounted adjustable rail system (Vevo Imaging Station) that allowed steerable and hands-free manipulation of the ultrasound transducer.

### 4.4. Isolation of Mononuclear Cells and Flow Cytometry

Immune cells were isolated from the peripheral blood, heart, and spleen as described previously [5,6,41,42]. Isolated cell suspensions from blood and other tissues were incubated for 1 h at room temperature in a cocktail of fluorophore-labeled antibodies to identify specific immune cell populations. Antibodies were used against: CD45 (605 NC, eBioscience clone # 30-F11), CD11b (Alexa Fluor 700, eBioscience clone # M1/70), Ly6C (PE Cy7, eBioscience clone # HK1.4), and F4/80 (eFluor 450, eBioscience clone #BM8). Lineage (Lin: PE anti CD45R/B220, BioLegend clone # RA3-6B2; PE anti CD90.2, BioLegend clone # 53-2.1; PE anti NK1.1, BioLegend clone #PK136; PE anti CD49b, BioLegend clone # DX5), Sca1 (FITC, eBioscience clone # D7), CD117 (c-Kit, PerCP-eFlour 710, eBioscience clone # 2B8), and CD115 (c-fms, APC, eBioscience, clone # AFS98). CD45 was used to identify leukocytes; CD11b and Ly6C were used to label monocyte populations, and F4/80 was used to characterize macrophages. Pro-inflammatory and patrolling monocytes were identified as CD45^+^CD11b^+^Ly6C^hi^ and CD45^+^CD11b^+^Ly6C^low^ cells, respectively. Inflammatory and reparative macrophages in the post-MI heart were identified within the CD45^+^CD11b^+^F4/80^+^ gate as Ly6C^hi^ or Ly6C^low^, respectively [18,43]. Data were acquired on LSRII flow cytometer (BD Biosciences) and analyzed with FlowJo software v10.0.6. The identified cell populations were normalized to the total CD45^+^ cell population. Splenic extramedullary hematopoiesis was evaluated by assessing populations of Lin^−^c-Kit^+^Sca1^+^ HSCs [16], Lin-c-Kit+Sca1- CMPs, [17], and Lin-CD115+c-Kitlow MDPs [16].

### 4.5. Isolation, Polarization, and In Vitro L-4F Treatment of Peritoneal Macrophages

Peritoneal macrophages were isolated by lavage from mouse peritoneum following 5 days of thioglycolate elicitation as previously described [44]. Peritoneal isolates from 6 mice were pooled and used for subsequent studies. After removal of non-adherent cells and following overnight incubation in culture media (DMEM, 10% FBS, 1% antibiotics), adherent macrophages were subjected to polarization for 4 h in serum-starved culture media. M1 polarization was induced by lipopolysaccharide (1 μg/mL) and interferon(IFN)-γ (4 ng/mL) treatment, whereas M2 polarization was induced by interleukin (IL)-4 (20 ng/mL) and IL-10 (20 ng/mL) [45]. Naïve cultured un-stimulated cells were considered as undifferentiated M0 cells. Following polarization, cells were washed and cultured in serum-free culture media for 2 h and treated overnight with either L-4F (50 μg/mL) or PBS. Similar studies were performed on the mouse macrophage cell line RAW 264.7 (ATCC TIB-71). At the end of the experiment, cells were washed and processed for RNA isolation and subsequent RT-PCR analysis.

### 4.6. RT-PCR Analysis

mRNA expression was quantitated using RT-PCR as previously described [5,46,47,48]. Briefly, TRIzol (Life Technologies) extracted total RNA was quantified using a NanoDrop 1000 spectrophotometer (Thermo Scientific). Total RNA (250 ng) was subjected to cDNA synthesis using the High Efficiency cDNA Synthesis Kit (Life Technologies). The levels of various mRNA transcripts were determined using Fast SYBR Green (Life Technologies) and gene-specific forward and reverse primer sets (Table 1) on a ViiA7 instrument (Life Technologies). 18s RNA expression was used to normalize mRNA expression using the ΔΔ*C*T comparative method.

### 4.7. Statistical Analysis

Analyses were performed using GraphPad Prism 7.0. All data are expressed as mean ± SD. Comparisons between two groups were done by using unpaired *t*-test for normally distributed variables. For comparisons of more than 2 groups, data were first assessed for normality using the Shapiro–Wilk test. For normally distributed data, comparisons among multiple groups were evaluated by one- or two-way ANOVA, with Tukey’s post-test for multiple comparisons. For non-normal distribution, the datasets were logarithmically transformed, and if normality was satisfied, analysis of variance and Tukey’s post-test were then performed. Changes in cardiac structure and function following L-4F treatment were evaluated by paired *t*-test analysis. A value of *p* < 0.05 was considered statistically significant.

## Figures and Tables

**Table 1:** RT-PCR primer sequences.

Gene Name	Gene ID	Forward Sequence	Reverse Sequence
GLUT-1 (SIca1)	20525	CGAGGGACAGCCGATGTG	TGCCGACCCTCTTCTTTCAT
HIF-1α	15251	GGGAGGACGATGAACATCAAG	TGGCCCGTGCAGTGAAG
HIF-2α (Epas1)	13819	ATGCCCTGGATTCGGAGAA	TGCCCCTTGGTGCACAA
HK2	15277	CCCTGCCACCAGACGAAA	GACTTGAACCCCTTAGTCCATGA
CCL3	20302	TTGGGGTCAGCGCAGATCTG	TCCCAGCCAGGTGTCATTTT
Arginase (Arg1)	11846	GCTCCAAGCCAAAGTCCTTAGA	CCTCGAGGCTGTCCTTTTGA
TNFα	21929	CAGCCGATGGGTTGTACCTT	GGCAGCCTTGTCCCTTGA
iNOS (Nos2)	18126	AGACCTCAACAGAGCCCTCA	GCAGCCTCTTGTCTTTGACC
CD206 (Mrc1)	17533	CCCAAGGGCTCTTCTAAAGCA	CGCCGGCACCTATCACA
18s rRNA	19791	CGAACGTCTGCCCTATCAACTT	ACCCGTGGTCACCATGGTA

## Supplementary Materials

Supplementary materials can be found at https://www.mdpi.com/1422-0067/21/10/3519/s1. Figure S1. L-4F enhances hematopoietic stem cell (HSC) abundance in WT naïve mice. A. Gating strategy for flow cytometric quantitation and corresponding group data of splenic HSCs, common myeloid progenitors (CMPs), and macrophage dendritic cell progenitors (MDPs) following L-4F (100 μg/day) or PBS treatment in WT naïve mice for 5 days. HSCs were identified as Lin^−^c-Kit^+^Sca1^+^ cells, CMPs were identified as Lin^−^c-Kit^+^Sca1^−^ cells, and MDPs were identified as Lin^−^CD15^+^c-Kit^lo^ cells. Data are depicted as number of cells per spleen, *n*= 3–4/group. * *p* < 0.05.

## Abbreviations

MI	Myocardial infarction
I/R	Ischemia reperfusion
HF	Heart Failure
apoA1	Apolipoprotein A-1
EDV	End diastolic volume
ESV	End systolic volume
EF	Ejection fraction
HSC	Hematopoietic stem cell
TNF	Tumor necrosis factor
CCL3	C-C motif chemokine ligand 3
iNOS	Inducible nitric oxide synthase
HIF	Hypoxia inducible factor
GLUT1	Glucose transporter 1
