# Supplementary material for: The Apolipoprotein A-I Mimetic L-4F Attenuates Monocyte Activation and Adverse Cardiac Remodeling after Myocardial Infarction
Source: Int J Mol Sci. 2020 May 15;21(10):3519. doi: 10.3390/ijms21103519 (PMC7279031; doi:10.3390/ijms21103519)
Supplement: Supplementary file 1 [file ijms-21-03519-s001.pdf]

Supplementary Materials

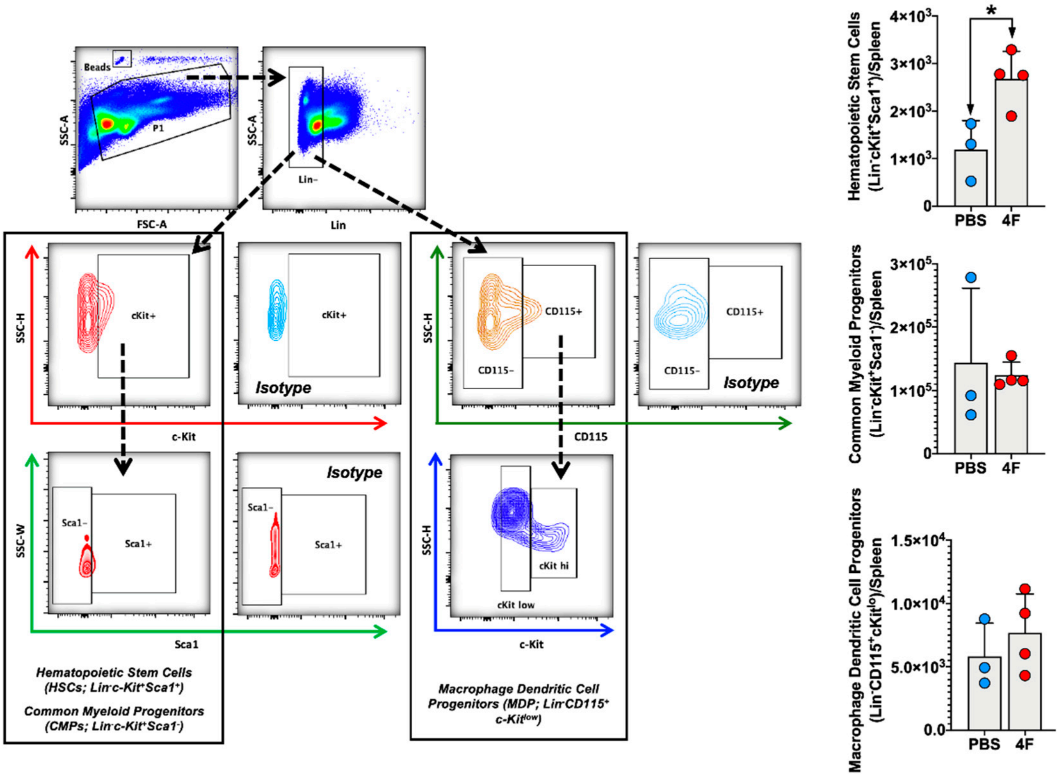

Figure S1. L-4F enhances hematopoietic stem cell (HSC) abundance in WT naïve mice. Gating strategy for flow cytometric quantitation and corresponding group data of splenic HSCs, common myeloid progenitors (CMPs), and macrophage dendritic cell progenitors (MDPs) following L-4F (100 µg/day) or PBS treatment in WT naïve mice for 5 days. HSCs were identified as $Lin^{-}c\text{-}Kit^{+}Sca1^{+}$ cells, CMPs were identified as $Lin^{-}c\text{-}Kit^{+}Sca1^{-}$ cells, and MDPs were identified as $Lin^{-}CD15^{+}c\text{-}Kit^{lo}$ cells. Data are depicted as number of cells per spleen, *n*= 3–4/group. * $p < 0.05$.
